# Evaluation of Internal Adaptation and Marginal Fit of Onlays Fabricated Using Computer-Aided Design (CAD)-Computer-Aided Manufacturing (CAM) and Three-Dimensional Printing Techniques: An In Vitro Study

**DOI:** 10.7759/cureus.40020

**Published:** 2023-06-05

**Authors:** Shiraz Pasha, Afreen Saleem, Muhammed Bilal

**Affiliations:** 1 Department of Conservative Dentistry and Endodontics, Sri Rajiv Gandhi College of Dental Sciences and Hospital, Bengaluru, IND

**Keywords:** micro-ct, cad-cam, digital impression, onlays, 3d printing

## Abstract

Background

This in-vitro study aimed to evaluate the internal adaptation, marginal fit, and applicability of digital intraoral impression techniques for onlays fabricated using computer-aided design (CAD)-computer-aided manufacturing (CAM) and three-dimensional (3D) printing techniques using a stereomicroscope and micro-CT scan.

Methodology

A total of 20 extracted mandibular first molars were selected for this study. The teeth were then divided into two groups. Onlay cavities were prepared involving the mesiobuccal cusp of the mandibular first molar in both groups. After preparation, both blocks were sent to the laboratory for fabrication of onlays using digital impressions (Shinning 3D scanner). Once the onlays were fabricated using CAD-CAM and 3D printing, a replica technique with monophase medium body impression material was used to assess the marginal fit and internal adaptation. The accuracy of internal adaptation was evaluated and compared using a stereomicroscope at 20× magnification. Measurements were taken at proximal margins, the inner axial wall, and the occlusal cavosurface area according to the Molin and Karlsson criteria. The same samples of both groups were studied for marginal fit using a micro-CT scan and values were recorded. The data collected were statistically analyzed using an independent Student’s t-test.

Results

Independent Student’s t-test results demonstrated that the mean thickness values of the material in the CAD-CAM group at occlusal cavosurface area, proximal area, and axial area were significantly higher when compared to the 3D printing group at p <0.001 and 0.005, respectively.

Conclusions

Internal adaptation and marginal fit of 3D-printed onlays were significantly lower than CAD-CAM onlays whereas the accuracy of 3D-printed onlays was significantly better than CAD-CAM onlays.

## Introduction

Ceramic onlays offer an excellent aesthetic solution for teeth that do not require full coverage restorations. Onlay restorations are required for broken or fractured teeth, cosmetic enhancement, decayed teeth, fractured fillings, and large fillings. Earlier, the conventional lost wax technique was used, but it produced casting defects and ill-fitting restorative margins which led to plaque retention, irritation, gingival trauma, and secondary caries. The advent of newer technologies such as digital impressions, computer-aided design (CAD)-computer-aided manufacturing (CAM) and three-dimensional (3D) printing have improved quality restorations with superior marginal fit and internal adaptation. Recent advancements in luting materials and procedures have given rise to a more conservative approach to ceramic onlay restorations rather than a full coverage restoration such as crowns which require finite retention forms [1].

Chairside and laboratory-based CAD-CAM systems have advanced which have provided a broader scope for clinical applications and products for dentistry. The changing trends and developments in the field of dentistry have made CAD-CAM and intraoral digital scanners an unavoidable alternative to conventional impression methods and techniques. With the introduction of a wide variety of digitalization tools and scanners, casting procedures have become obsolete.

Digital impressions are far superior to conventional impressions. The ease of clinician and patient comfort are some of the important factors. As patients with anxiety and gag reflex cannot tolerate conventional impressions, using digital impressions to substitute trays and materials is an ideal solution in these situations [2]. These are time efficient, simple, and equally accurate as the complex conventional impressions.

The quality of the marginal fit and internal adaptation are the key factors that determine the longevity and retention of indirect restorations. When onlay restorations are considered, the marginal fit and internal adaptation are substantial as their margins are exposed to different types of stresses such as physical, thermal, and mechanical [3]. Another important factor for the increased stability of all-ceramic restoration is its internal fit. The internal fit reflects the cement layer thickness [4].

Good marginal and internal fit can lead to long-term stability and retention of onlay restoration [4]. Increased periodontal pockets, gingival irritation, and bone deformities such as resorption can result from poor marginal adaptation [3]. Large marginal disparities can result in substantial wear and increased dissolution of the luting agent by chemical erosion. Margins that are well adapted can ease the process of removing excess luting agent before curing the cement, which helps in maintaining healthy periodontal tissues [5].

An advanced manufacturing technique known as 3D printing, often known as additive manufacturing (AM), is based on digitalized CAD design models. It uses standardized materials to produce customized 3D items using pre-programmed mechanized procedures. Rapid prototyping, layer manufacturing, AM, and solid free-form fabrication are additional terms for 3D printing. To produce 3D objects, it is a procedure in which various layers of material are added one at a time under computer supervision. The 3D model is divided into a number of thin layers, and the manufacturing machinery uses this geometric information to build each layer successively until the desired product is finished.

3D printing can provide better restoration accuracy compared to subtractive manufacturing techniques such as CAD-CAM. Another benefit is its low material loss. So, the present study aims to evaluate the internal adaptation, marginal fit, and applicability of digital intraoral impression techniques for onlays fabricated using CAD-CAM and 3D printing techniques using a stereomicroscope and micro-CT scan.

## Materials and methods

**Figure 1:**
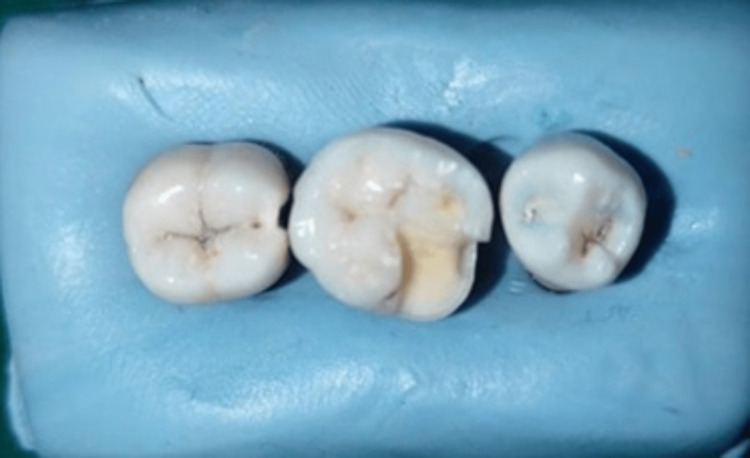
Onlay cavity prepared on the mandibular first molar.

**Figure 2:**
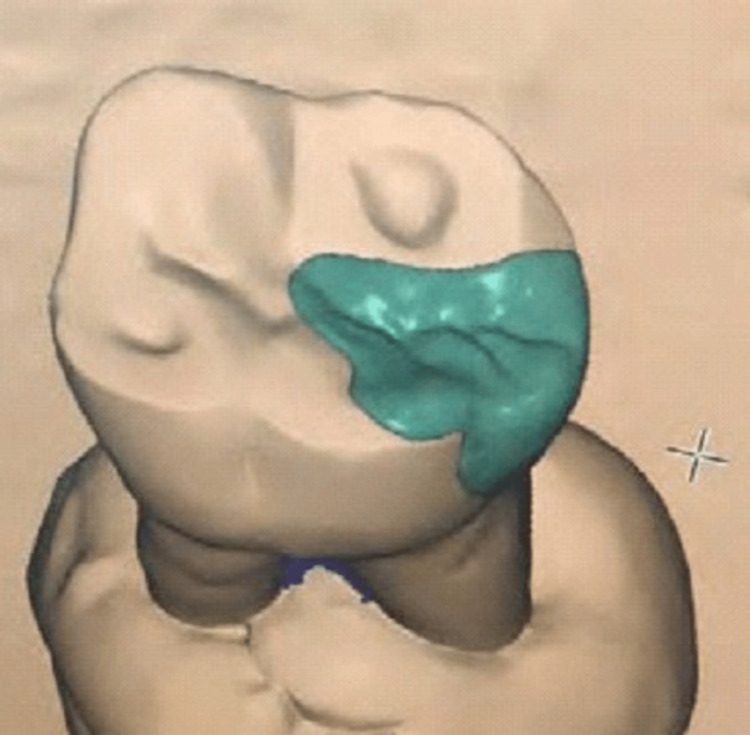
Digital scanning by a three-dimensional Shinning scanner.

A total of 20 extracted mandibular first molars without caries were selected for onlay preparation. All samples were mounted in rectangular-shaped addition silicone blocks along with the mandibular first premolar and mandibular second molar teeth to provide good proximal contact. Onlay preparation was done using 271 and 169L burs with high-speed air-rotor handpieces. The mesiobuccal cusp was included in all preparations. The cavity floors were prepared flat. With a fine diamond bur, the inner angles were rounded and smoothened. The mesiobuccal cusp was reduced by 2 mm, and the depth of the cavity was 2 mm at the central groove. The gingival floor depth was kept at 2.5 mm. All dimensions were standardized for all specimens (Figure 1). Samples were divided into two groups according to the fabrication method used (group 1: CAD-CAM, group 2: 3D printing). All samples were digitally scanned using a Shinning 3D scanner (Figure 2).

Digital impressions were made of each tooth using digital impression systems in both groups. In group 1, the data was transferred into CAD software using a CAD-CAM milling technique using the Amanngirrbach CAD-CAM machine. In group 2, the CAD data was saved in STL file format in the CEREC in lab SW15.0 CAD software. In group 2, 3D-printed onlays were manufactured with a 3D Shinning printer (Shinning 3D) in the laboratory. The CAD-CAM material used in this study was Aidite super perfect zirconia full ceramic. and the 3D printing material used in this study was a photopolymer resin.

**Figure 3:**
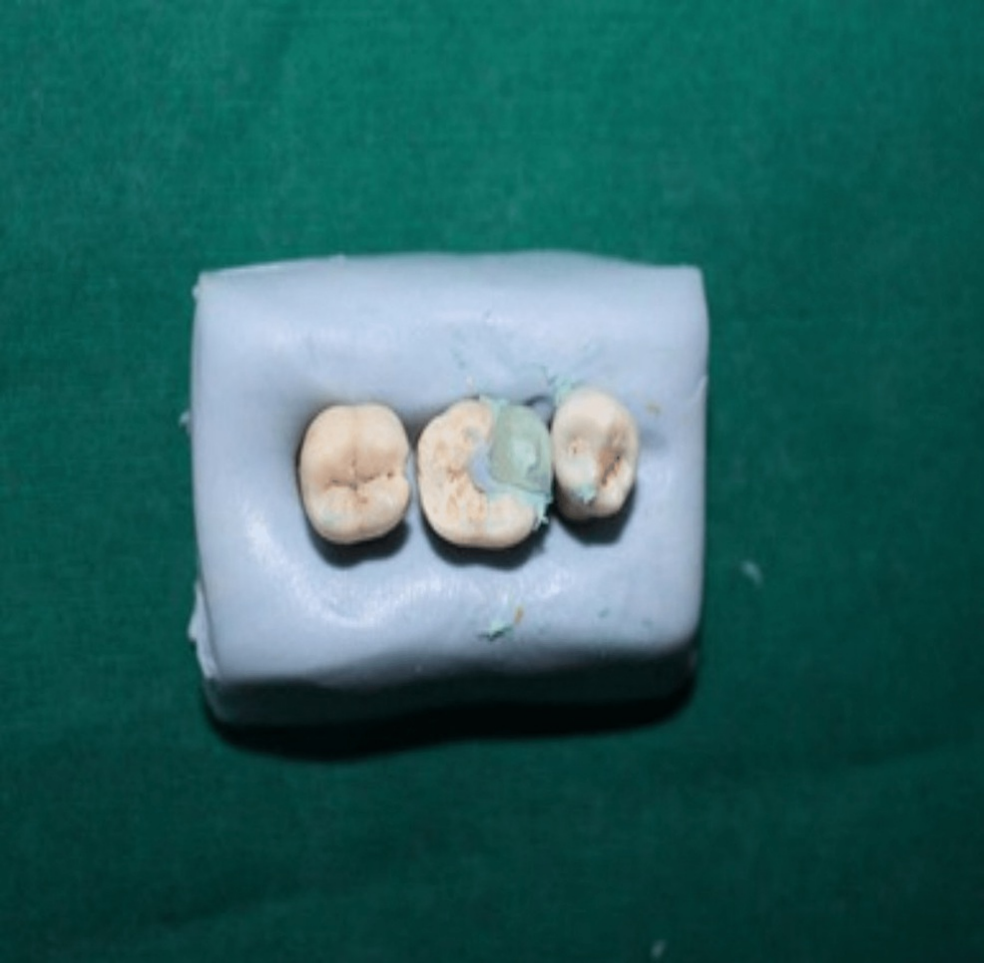
Three-dimensionally printed onlay placed on monophase medium body impression material.

**Figure 4:**
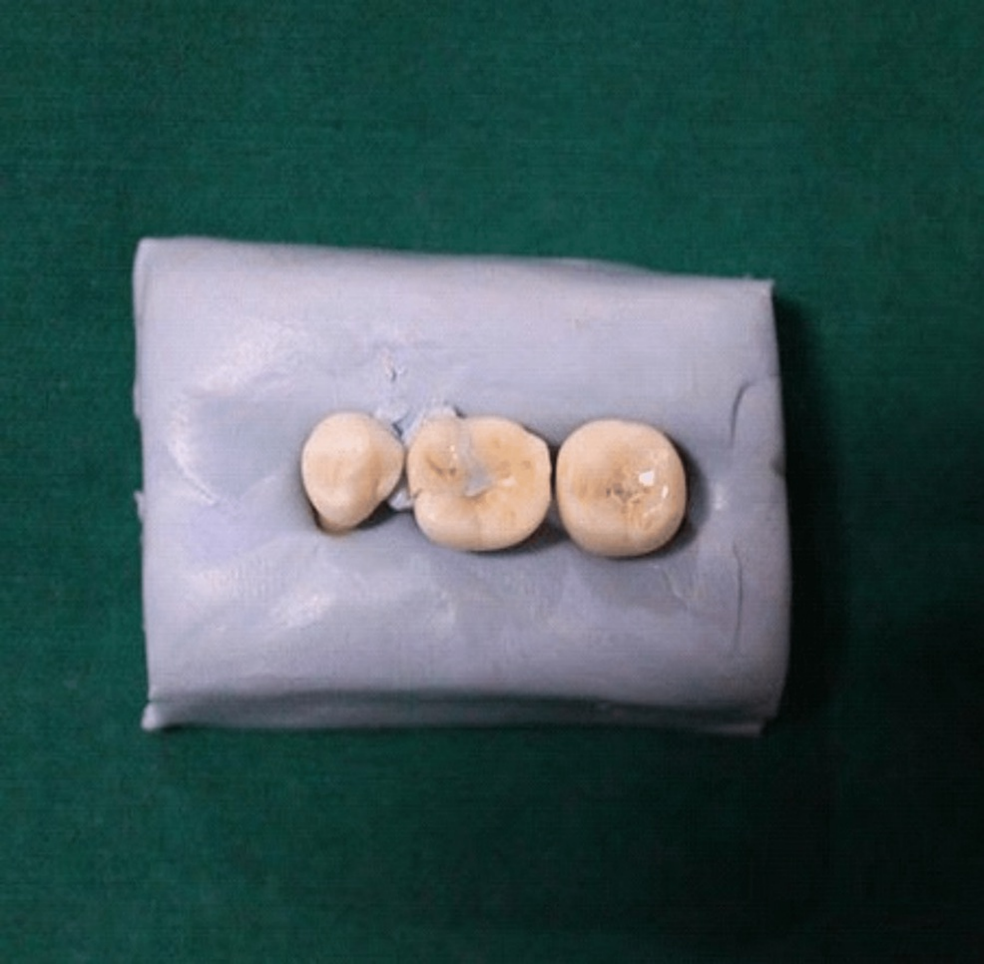
Computer-aided design (CAD)-computer-aided manufacturing (CAM) onlays placed on a light body impression material.

Once the onlays were fabricated, they were filled with light body addition silicone impression material and were fitted on respective teeth by applying finger pressure for three minutes (Figures 3, 4). After the material setting, onlays were gently taken out from the tooth and additional silicone mono-phase medium body with a different color was applied using finger pressure for one minute to give strength to the light body impression material.

The accuracy of internal adaptation was evaluated and compared using a stereomicroscope (Zeiss Discovery 20) at 10× magnification and 1,000 LP/mm. The replica technique described by Morlin and Karlsson was used to assess the marginal fit and internal adaptation. Measurements were taken at proximal margins, the inner axial wall, and the occlusal cavosurface area according to the Morlin and Karlsson criteria.

**Figure 5:**
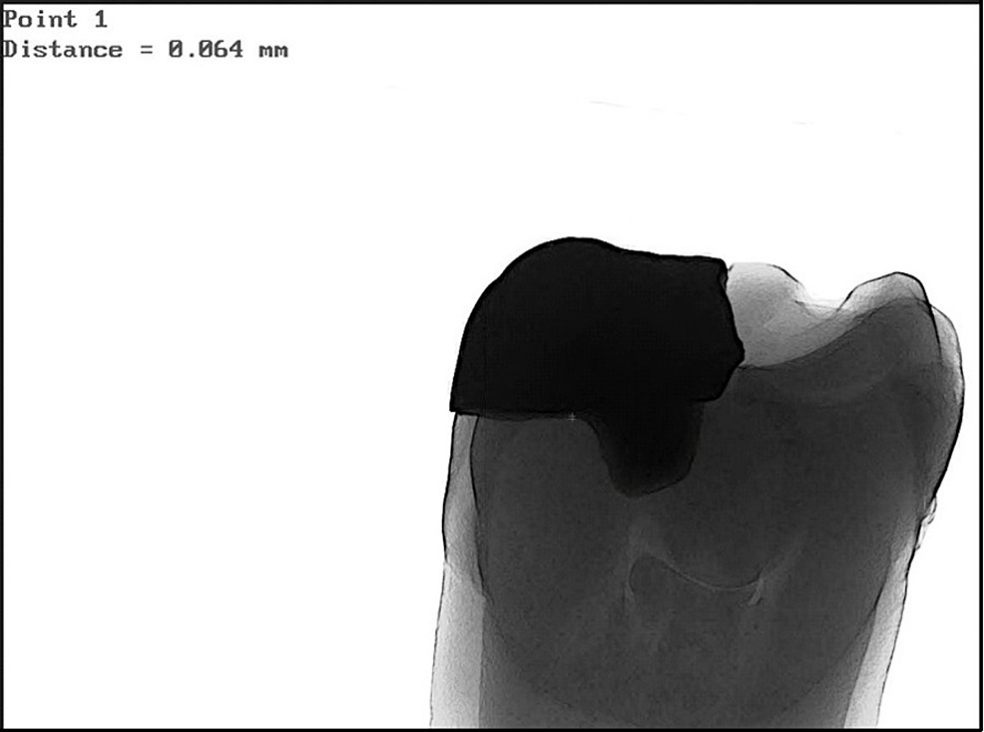
Micro-CT scan of three-dimensionally printed onlays at the proximal area.

**Figure 6:**
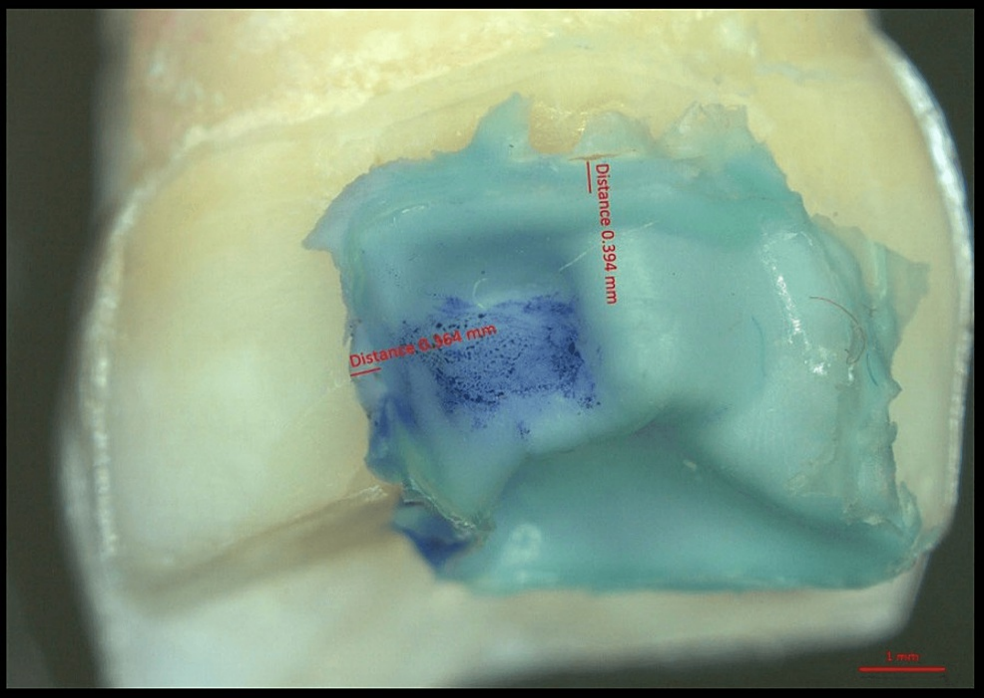
Stereomicroscopic image of Computer-aided design (CAD)-computer-aided manufacturing (CAM) onlays at the proximal and occlusal cavosurface area.

The same samples of both groups were studied for marginal fit and internal adaptation using a micro-CT scan (GE X-ray) and values were recorded (Figures 5, 6).

## Results

The comparison of the mean thickness of the putty material (in microns) by stereomicroscopic assessment between the CAD-CAM and 3D printing groups at different areas showed that the mean putty thickness of material in the occlusal cavosurface area measured for the 3D printing group (501.50 ± 85.27) was significantly lesser compared to the CAD-CAM group (981.80 ± 126.36). The mean thickness in the proximal surface in the 3D printing group was significantly lesser (445.00 ± 83.46) compared to the CAD-CAM group (788.90 ± 133.95) (p < 0.001). Similarly, in the axial surface, the 3D printing group showed significantly lesser thickness (213.60 ± 59.94) compared to the CAD-CAM group (359.20 ± 131.58) (Table 1).

**Table 1:** Comparison of the mean thickness of the putty material (in microns) by stereomicroscopic assessment between the CAD-CAM and 3D printing groups at different areas using independent Student’s t-test. CAD-CAM = computer-aided design-computer-aided manufacturing; 3D = three-dimensional; CI = confidence interval

Points	Group	N	Mean	SD	Mean difference	95% CI of the difference		t	P-value
						Lower	Upper		
Occlusal cavosurface area	CAD-CAM	10	981.80	126.36	480.30	379.02	581.58	9.963	<0.001*
	3D printing	10	501.50	85.27					
Proximal	CAD-CAM	10	788.90	133.95	343.90	239.05	448.76	6.861	<0.001*
	3D Printing	10	445.00	83.46					
Axial	CAD-CAM	10	359.20	131.58	145.60	49.54	241.66	3.184	0.005*

The comparison of the mean thickness of the putty material (in microns) by micro-CT assessment between the CAD-CAM and 3D printing group at different areas showed that the mean thickness of the putty material in the occlusal cavosurface area measured for the 3D printing group (93.20 ± 5.41) was significantly lesser compared to the CAD-CAM group (126.50 ± 20.96). The difference was statistically significant at p <0.001. The mean thickness in the proximal surface in the 3D printing group was significantly lesser (78.20 ± 7.63) compared to the CAD-CAM group (87.40 ± 10.28) (p = 0.03). Similarly, in the axial surface, the 3D printing group showed significantly lesser thickness (34.70 ± 8.04) compared to the CAD-CAM group (61.80 ± 10.34) (Table 2).

**Table 2:** Comparison of the mean thickness of the putty material (in microns) by micro-CT assessment between the CAD-CAM and 3D printing groups at different areas using independent Student’s t-test CAD-CAM = computer-aided design-computer-aided manufacturing; 3D = three-dimensional; CI = confidence interval

Points	Group	N	Mean	SD	Mean difference	95% CI of the difference		t	P-value
						Lower	Upper		
Occlusal cavosurface area	CAD-CAM	10	126.50	20.96	33.30	18.92	47.68	4.865	<0.001*
	3D printing	10	93.20	5.41					
Proximal	CAD-CAM	10	87.40	10.28	9.20	1.42	19.98	2.423	0.03*
	3D printing	10	78.20	7.63					
Axial	CAD-CAM	10	61.80	10.34	27.10	18.40	35.80	6.543	<0.001*

## Discussion

Functional and aesthetic restorations of extensively damaged posterior teeth need materials that should be biocompatible, mechanically stable for oral use, and tooth-colored. Ceramic onlays and partial ceramic crowns are restorations with one or more cusps being restored and are clinically accepted as alternatives to amalgam and cast gold restorations. However, these fail due to fractures predominantly [6].

Ceramic onlays are preferable to direct composite restorations when a posterior tooth is compromised by wide isthmus preparations because they offer an aesthetically pleasing, long-term alternative with a predictable level of clinical success. Because direct posterior composites have a restricted degree of polymerization conversion, which restricts their strength, ceramic onlays are stronger than direct posterior composite restorations and offer excellent physical properties.

In this study, onlay cavities were prepared because it is a less aggressive restoration than a crown as less tooth structure needs to be removed to place the onlay. In this study, onlay cavities involving the mesiobuccal cusp of the mandibular first molar were prepared. In a similar study by Eftekhar Ashtiani et al., a standard onlay was prepared according to the Hopp and Land criteria [7,8]. Correct preparation is important for the success of bonded onlays. Tooth preparation should include the retention and resistance forms, creating a design that facilitates laboratory fabrication of the restoration, which can simplify the cementation process and ease the adhesive bonding strategy of the restoration. A minimum thickness of around 1.8-2 mm is favorable for the restorative material. A few studies have reported that decreased restorative material thickness can make it weaker and more susceptible to fracture. However, recent studies have shown that few strong materials such as lithium disilicate and zirconia can perform better when thinner, or when they are bonded along with resin cement.

CAD-CAM has gained attention in producing dental prostheses. CAM fabrication is done either by a subtractive/reduction method or an additive method. Digital dentistry includes three main phases. The first phase is to digitize the physical shape of teeth and other oral structures using intraoral scanners or laboratory scanners. The second phase comprises digitally designing the restoration or oral prosthesis and is termed CAD. The third phase includes the transformation of the digital design made to a physical model by subtractive/reduction or additive rapid prototyping/3D printing [8].

In this study, an intraoral scanner (3D Shinning scanner) was used for digital impressions in the laboratory. Several studies have shown that the marginal fit and internal adaptation of the restorations was significantly better for those made with digital impressions than traditional impressions.

Direct fabrication of objects from 3D models using 3D printing or rapid prototyping involves building up each layer of the final product. The machining tools used and the characteristics of the material from which the restoration is made dictate how accurate milling is in subtractive technology. A harder material is less machinable and is likely to have structural flaws when it has finished being milled. For large complicated designs, it can be made via AM. Additionally, 3D printing is more cost-effective. This is because there is no material waste and any leftover material can be handled later. Printing numerous materials at once is made possible by AM technology. The field of dentistry is beginning to use 3D printing technologies. One of the key factors that decide the material strength and final outcome of a 3D-printed prosthesis is the build’s orientation or direction.

The CAD-CAM material used in this study was Aidite superperfect zirconia full ceramic. The color was white, 35% translucent, with a bending density of 1,300 Mpa, sintered density of 6 g/cm^3^, fracture toughness of 5 Mpa, and hardness of 1,250. The 3D printing material used in this study was a photopolymer resin (Shining 3D bio clear resin SG01). Photopolymers are compatible with numerous AM processes. In all of these processes, a resin is cured with light, then a full part prototype is built up layer by layer until completion. Shining 3D bio clear resin SG01 is a stable and highly standardized resin with good dimensional accuracy and excellent surface quality for showing details in prints. The color of the 3D-printed material was white.

In this study, after the fabrication of onlays, we assessed for internal adaptation and marginal fit of CAD-CAM and 3D printing. We used the replica technique as it is a more reliable and non-invasive method to determine the in vivo adaptation of the crown-to-tooth surface. Replication of the space between a tooth and its cast crown, using a light-body silicone supported by a heavy-body silicone, is a recognized technique to evaluate the quality of restoration. Impression techniques with impression material of low viscosity are popular methods for evaluating marginal discrepancies between crown and tooth. The replica technique is applicable to in vitro and in vivo measurements of precision. The replica technique’s advantage is in the fact that there is a small probability of damaging the sample in the process, which makes it a non-destructive method. A two-dimensional (2D) representation of the results is a slight drawback of this method. However, the majority of studies agree that, compared to other techniques, this technique provides an added possibility for false-proof and precise results. Therefore, it is an apt method for evaluating the accuracy of dental prostheses, which can also allow the quantification of the disparities on inner surfaces and marginal surfaces of the same. The replica technique has been previously used in studies to measure internal adaptations and marginal fit by Eftekhar Astiani et al. [7], Ahlholm et al. [9], and Nejatidanesh et al. [6].

Marginal fit is a key factor used in the evaluation of indirect restorations. Different methods have been used to measure in vitro the marginal and internal gap of a tooth-borne restoration (acceptable levels of 100 μm). However, X-ray microtomography (μ-CT) provides 2D and 3D images of the examined samples, this method provides close sections of the internal and marginal space. This was the only method that allowed both for precise identification of the critical distances and for a sufficient number of gap measurements.

The accuracy of internal adaptation was evaluated and compared using a stereomicroscope at 20× magnification. Measurements were taken at proximal margins, the inner axial wall, and the occlusal cavosurface area according to the Molin and Karlsson criteria. The same samples of both groups were studied for marginal fit using a micro-CT scan and values were recorded. Micro-CT scan (GE X-ray) offers very high resolution (5-50 μm voxel). This method allows for quantitative evaluation and analysis of the restoration’s internal space and is non-invasive/non-destructive. In a similar study, Eftekhar Ashtiani et al. evaluated the internal adaptation using stereomicroscopic analysis and marginal fit by micro-CT scan [7]. Another study used X-ray micro-CT for measuring the marginal fit of ceramic crowns.

The results of this study were statistically significant with 3D-printed onlays than CAD-CAM onlays in both methods evaluated for marginal fit and internal adaptation. The comparison of the mean thickness of the putty material (in microns) by stereomicroscopic assessment between the CAD-CAM and 3D printing groups at different areas showed that the mean putty thickness of the material in the occlusal cavosurface area measured for the 3D printing group (501.50 ± 85.27) was significantly lesser when compared to the CAD-CAM group (981.80 ± 126.36). Micro-CT analysis results showed that 3D-printed onlays had better internal adaptation and marginal fit than CAD-CAM onlays. The comparison of the mean thickness of the putty material (in microns) by micro-CT assessment between the CAD-CAM and 3D printing groups at different areas showed that the mean thickness of the putty material in the occlusal cavosurface area measured for the 3D printing group (93.20 ± 5.41) was significantly lesser compared to the CAD-CAM group (126.50 ± 20.96).

In a similar study by Ahlholm et al. [9], the accuracy of the inlay/onlay restoration was evaluated based on technique (3D printing or milling). Root canal-treated molar teeth were selected, and inlay and onlay cavities of different forms were prepared. Using CEREC AC digital impressions were made and the data were used to make nanoceramic restorations using milling and 3D-printed composite restorations with Multijet 3D. Using X-ray microtomography 3D imaging scans, the accuracy was analyzed by measuring the marginal fit and internal adaptation. Using the replica technique with additional silicone impression, the internal fit was evaluated. They concluded that the accuracy of the Multijet 3D printing is almost similar to the milling technique in the fabrication of inlay and onlay restorations [9].

This study evaluated the internal adaptation by stereomicroscope and marginal fit by micro-CT scan. 3D-printed onlays had favorable and significantly better results when compared to CAD-CAM onlays in the proximal, axial, and occlusal cavosurface areas. The accuracy of 3D-printed onlays was significantly better than CAD-CAM onlays. This helps to prevent secondary caries and microleakage, thus improving the restorability of the tooth. Measurements were taken in the proximal, inner axial wall, and occlusal cavosurface area which was significantly lower for 3D printing. The applicability of digital intraoral impression techniques for 3D printing provided better accuracy than conventional impressions.

Considering the restricted literature on the use of 3D printing technology for the fabrication of onlay restorations and the possible negative effects of this new method on the fabrication process, more studies with vast samples are required to evaluate the efficacy of 3D printers in terms of the dimensional accuracy of these restorations. Examining the results of materials science about 3D printing materials, the question of whether permanent dental restorations may be created by a 3D printer can be answered. Dental prostheses that are placed permanently in the mouth have significant biocompatibility needs. The usage of materials for definitive restorations must be able to tolerate both significant mechanical stress and the numerous chemical processes occurring in the oral cavity. To prevent bacterial deposits (plaque), the materials must have a smooth surface and not emit any toxic compounds in the mouth. Furthermore, a viable and cost-effective production method that can guarantee precision in the micrometer range must be accessible.

When comparing with milling systems which are in clinical use for quite a few years, presently there are no commercially available 3D printing materials or systems which are suitable, economical, biologically inert, and approved for use in permanent dental restorations such as inlay/onlay manufacturing. This must be taken into consideration when assessing the possibilities of 3D printing in the field of restorative dentistry. This study only assessed the marginal fit and internal adaptation. The material aspect, its biocompatibility, mechanical properties, and simulation of the oral environment were not studied. Future studies are required for assessing all other parameters.

## Conclusions

Within the limitations and conditions of this study, it can be concluded that the internal adaptation and marginal fit of 3D-printed onlays were significantly higher than CAD-CAM onlays. This can facilitate the prevention of secondary caries and microleakage, thereby improving the restorability of the tooth and the longevity of the restoration. The accuracy of 3D-printed onlays was significantly better than CAD-CAM onlays. Measurements taken in the proximal, inner axial wall, and occlusal cavosurface areas were significantly lower for 3D printing, highlighting that 3D-printed onlays were more accurate.

## References

[1] Goujat A, Abouelleil H, Colon P, Jeannin C, Pradelle N, Seux D, Grosgogeat B (2019). Marginal and internal fit of CAD-CAM inlay/onlay restorations: a systematic review of in vitro studies. J Prosthet Dent.

[2] Mangano F, Gandolfi A, Luongo G, Logozzo S (2017). Intraoral scanners in dentistry: a review of the current literature. BMC Oral Health.

[3] Khocht A, Zohn H, Deasy M, Chang KM (1996). Screening for periodontal disease: radiographs vs. PSR. J Am Dent Assoc.

[4] Guess PC, Vagkopoulou T, Zhang Y, Wolkewitz M, Strub JR (2014). Marginal and internal fit of heat pressed versus CAD/CAM fabricated all-ceramic onlays after exposure to thermo-mechanical fatigue. J Dent.

[5] Alajaji NK, Bardwell D, Finkelman M, Ali A (2017). Micro-CT evaluation of ceramic inlays: comparison of the marginal and internal fit of five and three axis CAM systems with a heat press technique. J Esthet Restor Dent.

[6] Nejatidanesh F, Shakibamehr AH, Savabi O (2016). Comparison of marginal and internal adaptation of CAD/CAM and conventional cement retained implant-supported single crowns. Implant Dent.

[7] Eftekhar Ashtiani R, Nasiri Khanlar L, Mahshid M, Moshaverinia A (2018). Comparison of dimensional accuracy of conventionally and digitally manufactured intracoronal restorations. J Prosthet Dent.

[8] Heymann HO, Bayne SC, Sturdevant JR, Wilder AD Jr, Roberson TM (1996). The clinical performance of CAD-CAM-generated ceramic inlays: a four-year study. J Am Dent Assoc.

[9] Ahlholm P, Sipilä K, Vallittu P, Kotiranta U, Lappalainen R (2019). Accuracy of inlay and onlay restorations based on 3D printing or milling technique - a pilot study. Eur J Prosthodont Restor Dent.

